# Comment on “Dermatitis herpetiformis in an African woman”, the importance of direct immunofluorescence assay

**DOI:** 10.11604/pamj.2020.36.196.18397

**Published:** 2020-07-20

**Authors:** Alice Verdelli, Marzia Caproni

**Affiliations:** 1 Department of Surgery and Translational Medicine, Section of Dermatology, University of Florence, Florence, Italy,; 2 U.O. Dermatology I, P.O. Piero Palagi, USL Toscana Centro, University of Florence, Florence, Italy

**Keywords:** Dermatitis herpetiformis, linear IgA bullous dermatosis, direct immunofluorescence

## To the editors of the Pan African Medical Journal

**Figure 1:**
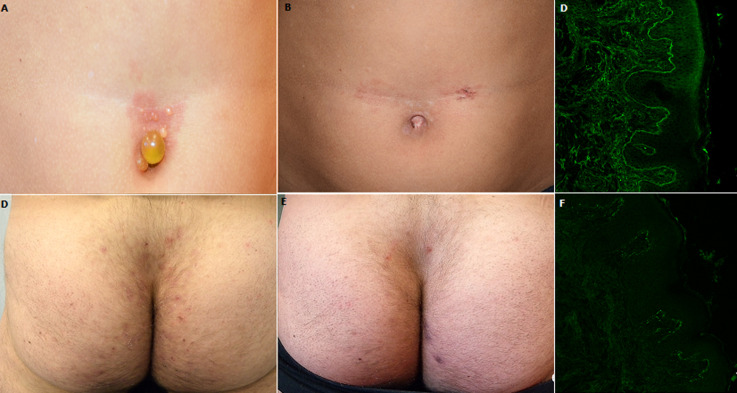
(A) linear IgA bullous dermatosis characterized by tense blisters grouped in the periumbilical area in a 10-year-old male; (B) linear IgA bullous dermatosis: hyper-pigmented maculae after treatment with dapsone in a 10-year-old male; (C) linear IgA bullous dermatosis: direct immunofluorescence on perilesional skin shows a linear IgA deposit along the dermo-epidermal junction; (D) post-bullous erosions in the gluteal region in a 32-year-old patient with dermatitis herpetiformis; (E) resolution of skin lesions in a patient with dermatitis herpetiformis after a 4-month gluten-free diet; (F) dermatitis herpetiformis: direct immunofluorescence on perilesional skin shows granular immunodeposits at the dermal papillae

We read with great interest the recent article by Machona MS *et al*. [1] about a 30-year-old female with a long history of itch and skin eruption associated to abdominal pain, nausea and vomiting. At clinical examination, the patient showed generalised, symmetrical polymorphic skin lesions located on the trunk, buttocks, extensor surface of the lower limbs and upper limbs. According to skin morphology and histopathology, a diagnosis of dermatitis herpetiformis (DH) with suspected celiac disease (CD) was made. The patient underwent a gluten-free diet (GFD) associated to dapsone treatment, with the improvement of both signs and symptoms in the follow-up period. In our opinion, the diagnosis in this case could be consistent with linear IgA bullous dermatosis (LABD) more than DH, and the clinical and histopathological examinations cannot be considered criteria sufficient enough to establish a diagnosis of DH. Clinically, the patient presented tense blisters in a pearl necklace-like arrangement on the extremities. These lesions are morphologically consistent with LABD more than DH [2,3]. Moreover, the post-inflammatory hypo- and hyper-pigmented macules on the abdomen can be found in LABD, while they are not typical of DH, whose lesions usually clear without post-inflammatory dyschromia (Figure 1).

Histopatological examination showed a sub-epidermal bulla with eosinophilic inflammation which are typical of an autoimmune bullous disease such as LABD [4]. Instead, the accumulation of neutrophils at the papillary tips (microabscesses), usually found in DH, are missing. Patients with LABD as well as DH dramatically respond to dapsone and treatment efficacy could not be considered as an adjuvant criteria for DH diagnosis [5]. According to DH diagnostic algorithm [6], in all the patients with clinical and/or histopathological findings suggestive for DH, a biopsy of perilesional skin for direct immunofluorescence (DIF) should be performed. DIF remains the gold standard for the diagnosis of DH, showing granular immunodeposits at the dermal papillae and/or along the basement membrane (Figure 1) [6,7]. Unfortunately, in this case it was not done. Moreover, IgA anti-tTG antibodies, which are considered the most sensitive and specific serologic investigation in patients with a suspected DH [6], should be collected. They were not dosed in this case. To exclude a CD, an endoscopy was performed but it did not reveal inflammatory changes. Even if a quarter of DH patients had normal small bowel villous architecture, in the long term, an increased density of gamma/delta intraepithelial lymphocytes can be found [8]. According to the most recent guidelines, duodenal biopsy could be avoided if immunopathological results are consistent with DH [9,10].

To conclude, the diagnosis of autoimmune bullous diseases is not easy and requires specific examinations. A correct early diagnosis is essential to provide adequate treatment. Since DH patients, contrary to LABD patients, need a lifelong GFD, we suggest to re-evaluate this case in order to make a correct diagnosis and to set the most appropriate long-term treatment.
